# Book Review

**Published:** 1937

**Authors:** 


### Reviews of Books

**Original Papers of Richard Bright on Renal Diseases.** By A. Arnold Osman, D.S.C., F.R.C.P. Pp. xvi., 172. Illustrated. London: Oxford University Press. 1937. Price 21s.—The subject-matter of Bright's famous work on renal disease needs no introduction to a medical public, but the comparative inaccessibility of the original papers has prevented the great majority of medical students and practitioners from making a personal study of them. For this reason Dr. Osman has, in the small volume under review, collected the three papers, and that portion of Bright's Gulstonian Lectures which deals with renal disease, and to this he has added an appendix in which the histological structure of three of the kidneys actually described by Bright is discussed and illustrated by photo-micrographs. In the first and most famous of the papers are included excellent reproductions of Bright's five beautiful hand-coloured plates of the kidneys he was discussing. The whole makes an attractive addition to our bookshelves, and Dr. Osman deserves the thanks of all medical men and women for providing this easy means of becoming acquainted with the writings of one of our most illustrious physicians. In these days of extensive clinical and pathological research in renal disease, when even those who are making a special study of this subject are apt to be confused by the multiplicity of terms and the enormous amount of detailed investigation, a perusal of these papers cannot but have a salutary and an encouraging effect. Above all, however, the volume is recommended on the grounds of the absorbing interest provided by an insight into the stages by which Bright developed that masterly conception of nephritis which has stood the test of time and made him rightly famous.

**A Social Problem Group?** Edited by C. P. Blacker, M.C., M.D., M.R.C.P. Pp. viii., 228. Oxford University Press (Humphrey Milford). 1937. Price 15s.—As this small book is of more than ordinary interest to both medical and lay publics it may assist the doubtful purchaser to indicate its contents. Edited by Dr. C. P. Blacker, the General Secretary of the Eugenics Society, it consists of a foreword and final summary by Mr. D. Caradog Jones, an introduction by the Editor, and eight articles on mental deficiency, mental disorder, epilepsy, inebriety, prostitution, recidivism, neurasthenia and unemployment, and the social problem group as a public charge—the whole being considered from the standpoint of the query at the end of the title. The authors of these several articles, not all of the medical profession, are men and women of recognized standing, sufficiently familiar with their subjects to make their reasoned and reasonable deductions of more than average interest. That this interest is of medical as well as of politico-social significance is obvious. The individuals who collectively or separately make up this social problem group repeatedly find their way into hospital, consulting-room and private practice. They always have done and apparently always will do. We fail to cure their condition, as we also do to prevent it, and hence the Social Problem Group. Something of these doubts seems to exist in the minds of the authors, for in his foreword Mr. Caradog Jones says "that the book is intended to raise questions in the mind of the reader; that to some of these questions the monographs themselves supply the answers, but that more questions have been asked than the authors lay any claim to have settled." That a perusal of the book does indeed cause the reader to ask questions is undoubted, but he need not read the book for the impetus: the questions arise of themselves; they always have done and always will do. Some more of them—but a mere fraction—may well be asked here. Most, if not all, medical men would attribute the phenomena of mind and its disorders to the constituent elements of the brain and nervous system rather than to Acts of Parliament. Why then are such problems as mental insufficiency, mental disorder, and many of the social problems arising therefrom governed and controlled by a miscellaneous assortment of Parliamentarians elected on the biologically erroneous basis of adult suffrage? Again, one of the authors says that "epilepsy is a wide term, probably covering a group of diseases." So is "coughing," but surely no one would regard coughing as a "group of diseases." It is a symptom of many disorders and even more diseases. Should we then also regard epilepsy as a symptom, and, if so, a symptom of what? Of both general and (on account of its author) local interest is Dr. C. W. J. Brasher's article on "Inebriety," wherein the same question arises. Is inebriety a disease, a habit, or a symptom ? "Alcoholism might thus prove to be only one of several aliases whose hereditary diathesis can only be expressed by some general term, such as want of mental balance." If this be the correct answer to the query, the further one arises—to what is this want of mental balance due ? If to habit it can be eradicated, but if to an ill-developed brain then its victims move in a vicious circle as inescapable as death itself. "What exactly do we mean by prostitution ? " asks Mrs. Neville Rolf. She supplies an excellent definition, but one that like even the best of definitions can be cavilled at. But is not prostitution, amateur or professional, a sign of life itself ? Are there not two great biological laws governing that life—the law of self-preservation and of reproduction of the species ? If a human female, condemned by the exigencies of the first law, finds herself compelled to use the second to fulfil the first, is she necessarily a member of the submerged tenth, the sub-cultural group, the a-social group, or whatever may be the polite piece of camouflage in vogue at the moment ? Are not the laws of Nature as compelling in the West End as in the East ? Notwithstanding his wisdom Solomon at least seems to have found them so. In this book bristling with problems there is much internal evidence that the authors themselves realize that a too close inspection of the trees may neglect the wood. The literature of both embryology and teratology teems with numerous examples of a failure of human bodily development. May not similar failures characterize, to a lesser or greater degree, man's most human feature, his own brain ? May not the mental defectives, the gross epileptics, the confirmed recidivists, the chronic alcoholics, the premature dements, the a-sexual commercial male and female prostitutes, and the ill-begotten progeny of the lot, be really victims of an ill-conditioned, ill-developed, ill-controlled cerebral and nervous system ? If so, prevention is the only cure, but *that* tide is not yet on the flow in England, and the distinguished authors of this book, though they put before us ably and well the many problems permitted by the maudlin sentimentality of to-day, suggests neither cause or cure nor mode of prevention. Readers of the printed lines of this book will find much to interest them. Those who read between the lines as well will find even more, and both will ask questions, but that, we are assured, is one of the objects of the book. Judged by such a standard—its own—the work is a striking success.

**A Doctor at Work and Play.** By S. H. SNELL, M.D., B.S., D.P.H. Pp. viii., 351. Illustrated. London: John Bale, Sons and Curnow Ltd. 1937. Price 12s. 6d.—Dr. Snell, who passed away immediately before the publication of this work, was a man of exceptionally good physique and abounding energy. He delighted in all forms of sport—cycling, golf, hunting, fishing and motoring—and in spite of the claims of a busy general piactice he attained a high degree of efficiency in all of them. He relates his sporting experiences in a peculiarly racy manner, and his store of witty and amusing tales is apparently inexhaustible. His medical experiences (and we could wish there were more of them) will be of particular interest to men who qualified in the nineties, and his chapters on hobbies, such as the keeping of goats, dogs, chickens, and on farming generally, will be read with appreciation by members of the medical profession who are contemplating or enjoying retirement. Dr. Snell has much to say about his travels in the United States of America, on the Continent of Europe, and in Scotland and Ireland, but he is at his best in his account of Bournemouth and its immediate neighbourhood, a district to which he was much attached, and in which he spent the last years of his life.

**The Human Foot.** By DUDLEY J. MORTON. Pp. xiv., 244. Illustrated. New York: Columbia University Press. 1935. Price 15s.—We are presented with an easily read book which is admirable from many points of view beyond the mere printing, illustrations, and selected list of references. The text is planned as though the comparative anatomist speaks first. He gives us a simply expressed and interesting history of how bodily progression was first evolved from the very nursery of organic life; how our static feet were adapted from the prehensile ones of monkeys and apes; and how bones, muscles and ligaments became structurally altered by functional requirements. The physiologist continues by considering our upright posture; our feet in stance, balance, and their mechanics in motion from walking to running. The orthopædic surgeon describes the proper examination and diagnosis; the primary causes of foot disorder, and their treatment with several remedial devices (which are protected against unauthorized commercial exploitation!). The general practitioner is now stirred to realize that he should treat disabilities of the foot as part of his province, and not relegate them to the osteopath, the chiropodist, or the boot-maker.

He will find in this book remarks upon "foot-wear," arch supports, exercises and other remedies; sex, age, ways of walking, constitutional disorders, etc., are cognate topics, even to the point of corns and shrunken socks; and X-ray examination of the feet is emphasized and illustrated.

**Principles of Diagnosis, Prognosis and Treatment: a Trilogy.** Second Edition. By ROBERT HUTCHISON, M.D., LL.D., F.R.C.P. Pp. viii., 54. Bristol: John Wright & Sons, Ltd. 1937. Price 3s. 6d.—Dr. Hutchison's trilogy performs a salutary service in these days of "Mechanized Medicine," when patients, and alas too often doctors also, are apt to act as though diagnosis were simply a matter of a penny in the slot of the appropriate X-ray machine and pathological department, when prognosis has become a matter of mere reference to statistical tables, and when we have sold our therapeutic birthright to the patent medicine manufacturers. It reminds us that we are still "doctors," and that good doctoring must rest on certain basic fundamental principles. These principles are clearly set forth in this little volume, which contains nothing new, but also nothing that will not bear re-telling many times. It could be read and re-read with advantage by every student and by the vast majority of practitioners.

**A System of Clinical Medicine.** Tenth Edition. By T. D. SAVILL, M.D., edited by A. SAVILL, M.D., and E. C. WARNER, M.D., F.R.C.P. Pp. xxviii., 1,114. Illustrated. London: Edward Arnold & Co. 1936. Price 28s.—Savill's *System of Clinical Medicine* differs from the usual text-book of medicine in that the customary "systematic" approach to disease has been entirely discarded, and the reader is introduced to medicine from the point of view of symptomatology. Every disease is considered starting with a hypothetical patient with certain symptoms and physical signs. The description of the disease in question is then followed by a detailed consideration of the differential diagnosis. A large part of the book thus becomes an index of clinical diagnosis. As such it is probably of greater value to a practitioner than is the usual text-book. The student will probably find that the novel approach is of great value in fixing in the mind the main features of a disease after it has been read in an orthodox systematic treatise. It should be of great value to students also in stressing the importance of a careful and accurate consideration of symptomatology in the diagnosis of disease. This, the tenth edition, has been carefully revised with the aid of numerous collaborators without altering the original plan and arrangement. It is well printed and illustrated, and space has been saved by printing the description of less common conditions in small type.

**Medical Diagnosis: Some Clinical Aspects.** By S. LEVY SIMPSON, M.D., M.R.C.P. Pp. xii., 244. Illustrated. London: H. K. Lewis & Co. Ltd. 1937. Price 10s. 6d.—This small book of just over 200 pages gives in a readable way a synopsis of the physical signs and symptoms which the author considers "essential for general medical diagnosis," and for those surgical diagnoses which should not be missed "by a good physician." After an introduction which contains good, sensible, fatherly advice we have a dozen chapters in which the diagnosis of a wide range of medical subjects is considered in a comprehensive fashion. For example, the chapters on the renal system and blood diseases are each treated in over twenty divisions, while the chapter on nerve diseases has almost twice as many sections. For the most part clinical bedside diagnosis is aimed at, but the importance of special microscopic and chemical examinations, etc., is indicated when necessary, and there are many useful tables of pathological findings in these cases. The scope of the book does not allow room for differential diagnosis, and no prognosis nor treatment is discussed. The book is written chiefly for the general practitioner, and although certain sections may appear elementary, other parts contain a résumé of rare diseases and newer methods of diagnosis which should prove very helpful. One can say, perhaps, there is nothing in the book but what can be found in an up-to-date text-book of medicine, but this little book deals with diagnosis (the sheet-anchor of general practice) and with this only. It is written clearly and concisely, and can be relied upon for exactness, and should save a good deal of time and mental effort for the busy doctor.

**An Introduction to General Practice.** By E. KAYE LE FLEMING, M.D. Pp. viii., 150. London: Edward Arnold & Co. 1936. Price 5s.—Although this book is described as *An Introduction to General Practice*, there is much in it of interest and value to an older practitioner. There are very few points of difficulty which are not cleared up in its pages. The chapters on the General Medical Council and the "Doctor and Patient " would, if read by the general public, remove much misunderstanding, and the public might then realize how much is done for its protection. We recommend this book to all seeking information on commencing practice, and hope it may be widely read by the public as well as the doctor.

**Contributions to Clinical Practice. Vol. I.** By MEMBERS OF THE STAFF OF SOUTHEND GENERAL HOSPITAL ; edited by T. ROWLAND HILL, M.D., M.R.C.P. Pp. viii., 208. Illustrated. London : Southend General Hospital. 1936. Price 12s. 6d.— This volume contains articles contributed by members of the staff from all departments of the Southend General Hospital. There are two interesting papers on gastric surgery by Mr. Rodney Maingot, while Mr. Aleck Bourne discusses the indications for induction of labour. Other papers cover the whole range of medicine and surgery from " Hereditary Hæmorrhagic Telangiectasia " by Dr. L. T. Bond to " Intracranial Tumours " by Dr. Rowland Hill. At the end of the book are reports of several unusual and interesting cases. The subjects described are dealt with almost entirely from the personal viewpoint of the author, and references to the rest of the literature are scanty. While the book is of interest in thus portraying the results of each of the authors' practical experience of his subject, one cannot help feeling that the majority of the articles add little or nothing to our present knowledge. It would appear that those which are of value from this point of view should have been published through one of the more usual channels in the already existing medical periodicals, where they would reach a larger public and be more readily accessible. In view of the vast amount of medical literature with which we are at the moment inundated, it is rather doubtful if the publication of such a volume as this is justified. The book is well produced— plentifully illustrated by X-ray photographs and diagrams— and the publishers are to be congratulated on the general excellence of the type.

**Vade Mecum of Medical Treatment.** By W. G. SEARS, M.D., M.R.C.P. Pp. viii., 368. London : Edward Arnold & Co. 1937. Price 10s. 6d. — This book provides in a convenient and handy form, an account of the treatment of diseases most commonly met with in General Practice. The various treatments advocated are arranged in alphabetical order and easy reference is thus assured. All these treatments appear to be along sound lines, thoroughly authenticated by experience. The directions are quite complete, whilst the writer is careful to warn his readers in the preface that accurate diagnosis must take precedence over any treatment prescribed. Consequently we find that certain methods and points of diagnostic importance have had to be included. Many useful prescriptions are given and the details of the vitamin content of common foods (p. 348) are also convenient. This book is well produced and adequately indexed. It will certainly prove most useful to hospital residents and senior students, whilst practitioners will find a fund of information most concisely expressed.

**Diseases of Children.** By F. M. B. ALLEN, M.D., M.R.C.P. Seventh Edition. Pp. vii., 329. London : Messrs. Baillière, Tindall & Cox. 1937. Price 4s. 6d.—This little book has been re-written and brought up to date with present edition. In a small space there is a very good summary of diseases of infancy and childhood, with a useful discourse on treatment. The section on Infant Feeding and management of premature infants has been dealt with in some detail, as have also the other sections of special importance to the general practitioner, such as specific fevers and diseases of the respiratory tract. There is an excellent appendix, which gives the student handy and concise information, including many prescriptions.

**Sick Children.** By D. H. PATERSON, M.D., F.R.C.P. Second Edition. Pp. iv., 600. Illustrated. London : Cassell & Co. Ltd. 1937. Price 12s. 6d.—The appearance of a second edition of this book will be welcomed by general practitioners and students, for here they will find up-to-date details of the diagnosis and treatment of sick children. The recent advances in the knowledge of the anæmias are fully dealt with, and the footnotes giving references to articles of special interest seem an excellent idea. The feeding tables have been modernized, and the recent examination papers given in the appendix will prove invaluable to students. The illustrations throughout are very good, and the book is well produced and may be safely recommended.

**Diagnosis and Non-Operative Treatment of the Diseases of the Colon and Rectum.** By G. SCHWARZ, M.D., J. GOLDBERGER, M.D., and C. CROCKER, M.D. Pp. x., 540. Illustrated. London : H. K. Lewis Ltd. 1937. Price 40s.—This book is written by a famous Vienna radiographer and two physicians, one in Carlsbad, and the other in New York. It is excellently produced and illustrated, the coloured pictures of sigmoidoscopic appearances being particularly good. The chapter on the X-ray examination of the colon is very good. An improved sigmoidoscope providing vision through a water medium, called the "deplicoptic procto-sigmoidoscope," is recommended. A distinction is drawn between two varieties of constipation, the *dyskinetic* (dyschezia of Hurst), in which the barium meals show stagnation in the ascending colon and the rectum, with spasm between, and the *hypokinetic*, in which the passage of fæces is very slow all along the colon. There is a good discussion of the various forms of colitis and their treatment. Another excellent article is that on the treatment of hæmorrhoids, whether by injection or by less drastic methods. The whole book is very sensible, and full of simple directions for treatment that can be carried out by any doctor and usually in the patient's own home.

**Charterhouse Rheumatism Clinic Original Papers, Volume I.** Pp. x., 203. London: Oxford University Press (Humphrey Milford). 1937. Price 15s.—The first paper in this volume, on "Pathogen Selective Culture and its bearing on the Classification and Aetiology of Chronic Rheumatic Disease," by H. Warren Crowe, should be studied by all who are interested in the rheumatic problem. The author stresses the importance of infection in all forms of rheumatism; but whether the ability of a micro-organism to resist the bactericidal powers of an individual's blood serum, apparently, in the author's view, because it is protected by phagocytosis, is sufficient evidence that that organism is in some part responsible for his rheumatic trouble, will perhaps not receive universal acceptance without a large series of controls in other conditions. The article, however, is of great interest and contains many shrewd observations. The second paper, by Harry Coke, is a description of the technique and practical applications of the Differential Sedimentation Test. Time alone will show whether the information imparted by this somewhat complicated procedure will be sufficient to cause it to supplant the simpler erythrocyte sedimentation rate. In the third paper Gilbert Scott again enunciates his view that spondylitis ankylopoietica is due to an infective focus in the sacro-iliac joints, a highly improbable supposition with little in its support. That the sacro-iliac joints are generally amongst the first of the "root" joints to be involved is undoubted, but the evidence that they actually constitute an initial focus of infection is far from conclusive. For treatment of this form of spondylitis the author advocates wide-field radiation. The manner of presentation of the papers leaves nothing to be desired and the illustrations are excellent.

**The Facial Neuralgias.** By WILFRED HARRIS, M.D., F.R.C.P. Pp. xii., 110. Illustrated. Oxford University Press (Humphrey Milford). 1937. Price 7s. 6d.—This monograph is really a supplement to Dr. Harris's earlier work, *Neuritis and Neuralgia*. Naturally a large section of the book is devoted to trigeminal tic. The author speaks from a vast personal experience, and the advice he gives, particularly on the technique of operation by injection, is invaluable to everyone who practises alcohol injections for this distressing malady. Harris's lateral approach offers a perfectly safe method, provided his words of warning with regard to the possible pitfalls in the operation are noted, for they are all capable of recognition in time so that no harm is done. The rest of the book is devoted to a description of other neuralgias, such as geniculate and glossopharyngeal tics, which, though comparatively rare, may be very puzzling. The whole subject of facial neuralgias and their differential diagnosis is admirably presented in a form that is both readable and practical. The book is well illustrated and contains two valuable anatomical diagrams of the trigeminal nerve connections in both their central and peripheral course.

**The Vegetative Nervous System.** By WULF SACHS, M.D. Pp. x., 168. Illustrated. London: Cassell & Co. Ltd. 1936. Price 15s.—A knowledge of the functions and anatomy of the vegetative nervous system is becoming more and more important in medicine, because the clinical applications of the numerous discoveries which have recently been made in this section of the nervous system are daily becoming more valuable in the elucidation of diverse and obscure clinical phenomena. In this volume the author describes in very clear language the various methods which should be employed for making as complete an examination as possible of this system. Furthermore, he describes in detail not only the anatomical, but also the physiological and pharmacological data of the vegetative nervous system, together with the numerous physiological and physical reflexes, finishing with a chapter on the general clinical significance of the system. This little book forms an excellent epitome in a very compact form of all this knowledge, and may be recommended to students and practitioners, for the information it contains is not easily found in ordinary text-books of medicine.

**An Introduction to Psychological Medicine.** By R. G. GORDON, M.D., D.Sc., F.R.C.P., N. G. HARRIS, M.D., B.S., D.P.M., and J. R. REES, M.D., D.P.H. Pp. x., 386. Illustrated. London: Oxford University Press (Humphrey Milford). 1936. Price 10s. 6d.—Books dealing with psychological themes are abundant, but we do not recall one that will fill such a gap so successfully as will this. Written for the medical student, it envisages a course of four or five lectures in psychology, three or four on psychopathology, five on the psychoneuroses, eight on the psychoses, and two on mental deficiency. Unfortunately, few medical students have the opportunity of receiving instruction to this extent, so that the book gives more than the present students are likely to require. Nevertheless, it will be of great value to those students who are particularly interested in psychological medicine. The matter has been selected with care and is well balanced, there being 74 pages on psychology, 56 on psycho-pathology, 84 on the psychoneuroses, 112 on the psychoses and 30 on mental deficiency. In addition, there is a chapter on legal aspects and also a bibliography. Treatment is not described in detail, as psychotherapeutic technique is a post-graduate study, the purpose of this book being the instruction of the student in the mind, psychopathological processes and the various clinical entities. The book fulfils its purpose admirably. Unfortunately, the type is set too close to be attractive, the pages being given an appearance of solidity, which may prevent some from reading the book. The bibliography is not quite up to date as regards the latest editions. The index is comprehensive.

**Elements of Orthopædic Surgery.** By N. ROSS SMITH, M.B., Ch.M., F.R.C.S. Pp. 246. Illustrated. Bristol: John Wright & Sons Ltd. 1937. Price 10s. 6d.—This short book describes the scope of orthopædic surgery, indicating what cases can be benefited by this specialized treatment. The author has refrained from giving technical details of difficult operations and has confined himself to the diagnosis and management of the commoner forms of crippling. In the fracture section he has described the modern advances in fracture treatment during the last ten years. The book is excellently produced, well illustrated, and very easy to read. It should be of great service to General Practitioners, Medical Students, Massage Students, or to anyone who wants a brief, uncomplicated summary of modern orthopædics.

**Practical Orthoptics in the Treatment of Squint.** By KEITH LYLE, M.D., M.Ch., M.R.C.P., and SYLVIA JACKSON, S.R.N. Pp. xvi., 212. Illustrated. London: H. K. Lewis & Co. Ltd. 1937. Price 12s. 6d.—The authors of this new manual of squint-training are on the staff of the Royal Westminster Ophthalmic Hospital, where the first orthoptic clinic in England was opened seven years ago. They have had opportunities of dealing with a large quantity of clinical material, comprising all types of squint and heterophoria, and in this book have treated the whole subject in a clear and essentially practical manner. The chapters are well arranged, and include instructions on the management and equipment of a clinic, the methods of examination, and the entire technique of fusion-training. The prospects of curing or improving the various types of squint are fully discussed, and very interesting tables are included, giving in detail the steps in treatment and the final results in a large number of cases completed at the clinic. One is impressed, not only by the high percentage of good results obtained on the whole, but also by the patience and skill which must have been expended by the various workers concerned. Ophthalmic surgeons will find especially interesting the chapters dealing with the principles of operation and with ocular torticollis. Although intended mainly as a practical manual for students training for the examination of the Orthoptic Board, it should be studied by all who are interested in modern methods of treating squint and heterophoria. It is an excellent and up-to-date presentation of the subject, and can be cordially recommended.

**Latent Syphilis.** Second Edition. By G. EVANS, D.M., F.R.C.S. Pp. xi., 158. Illustrated. Bristol: John Wright & Sons Ltd. 1937. Price 7s. 6d.—The second edition of this book differs in no material respect from the first, our criticism of which has evoked from the author the compliment of a preface devoted to answering it. But our opinion remains unshaken. He writes of a syphilis without tryponema, chancre, rash, gumma or Wassermann reaction, and maintains that it is, if not ubiquitous, at least responsible for the bulk of all chronic disease. As an example we may refer to his chapter on dysphagia with anæmia. Prominent features of this disorder are superficial glossitis (but never leukoplakia), sore throat, and cracks at the angles of the mouth: and in consequence a mistaken diagnosis of syphilis has very often been made. As a result all those who have made a particular study of the syndrome have devoted special attention to the question of syphilis, and all alike have agreed that there is no connection between the two. Yet the author persists in the opposite view. The argument that arsenic is beneficial is worthless: we are dealing with a simple secondary anæmia, which we might expect to be relieved by the administration of arsenic, but more simply by adequate doses of iron. The thesis that a latent syphilitic taint is responsible for a great deal of obscure chronic disease is one worthy of emphasis: in this book exaggeration has ruined it.

**Pathological Physiology and Clinical Description of the Anæmias.** By W. B. CASTLE, M.D., S.M., and G. R. MINOT, M.D., S.D., F.R.C.P. Pp. x., 206. Illustrated. London: Oxford University Press (Humphrey Milford). 1936. Price 15s.—To a generation accustomed to expect even their educational treatises presented in the most stimulating and attractive style, the first impressions made by this book are not entirely happy. The texture of the paper, the typography, the absence of chapters and of distinctive headings, and the lack of diagrams and illustrations combine to produce a monotonous effect calculated to deter all but the most persevering of scholars. Perseverance, however, will be well rewarded. The authors, whose work has played so large a part in revolutionizing our conceptions of the pathology and treatment of pernicious anæmia, have given us in this book a clear and concise account, not only of the various types of anæmia, but also of modern therapy in relation to them. Their theories are expressed logically and definitely, and redundancy is so firmly avoided that every sentence merits the closest attention. All the most modern work has been incorporated, making the book of special appeal to the hæmatologist and clinical pathologist, though there is perhaps a tendency to accept as established much that has been so recently worked out that it still requires confirmation. The elimination of obscure terms such as "leukanæmia" and "achrestic anæmia," the lucid exposition of "hæmolytic anæmias," the plea for the prolonged treatment of pernicious anæmia necessary to improve neurological lesions, the section on blood transfusion in all its aspects, and the unusually comprehensive bibliography are but a few of the features which combine to give us a work of outstanding merit and interest.

